# Calculation of reference intervals using an indirect approach from laboratory database

**DOI:** 10.1515/almed-2025-0088

**Published:** 2026-02-09

**Authors:** Victor Martin-Riera, Pablo Gabriel-Medina, Albert Blanco-Grau, Gonzalo Gonzalez-Silva, Yolanda Villena, Andrea Caballero-Garralda, Lydia Peris-Serra, Sarai Garriga Edo, Clara Sanz Gea, Fernando Moreno, Andrea Arias-García, Jaume Barallat, Celia Monteagudo-Lopez, Laura Conesa

**Affiliations:** Clinical Biochemistry Department, Drug Delivery and Therapy Research Group, Vall d’Hebron Research Institute (VHIR), Vall d’Hebron University Hospital, Barcelona, Spain

**Keywords:** adult population, indirect approach, laboratory tests, reference intervals

## Abstract

**Objectives:**

The establishment of reference intervals (RIs) for biochemical parameters is crucial for the accurate interpretation of laboratory test results and clinical decision-making. This study aims to evaluate the feasibility of determining RIs using an indirect approach, which analyzes routine clinical samples instead of recruiting healthy individuals, thus leveraging large datasets while applying strict statistical criteria to exclude pathological values.

**Methods:**

This is a prospective study in which patients fulfilling inclusion criteria were recruited from the routine primary care to achieve a high proportion of healthy individuals. Biochemical magnitudes as creatinine, esterified bilirubin, ferritin, and transferrin were analyzed. A patient exclusion protocol was implemented to minimize results suggestive of pathology but ensuring a sufficient sample size for the calculation of new RIs. The new ones were compared with the RIs used in the laboratory, provided by the manufacturer.

**Results:**

The comparison between the newly calculated RIs and the existing laboratory RIs did not reveal statistically significant differences. The indirect approach provided robust RIs while maintaining data quality and a sufficient sample size.

**Conclusions:**

The findings support the effectiveness of this indirect approach for RI determination, provided that rigorous data quality standards and adequate sample sizes are maintained. The adoption of these RIs in clinical practice could enhance diagnostic accuracy and optimize healthcare resource utilization.

## Introduction

In the context of clinical decision-making, reference intervals (RIs) serve as a valuable tool, facilitating the interpretation of analytical results and supporting the estimation that laboratory data underpins approximately 70 % of these decisions [1]. The term RIs is used to describe the interval of test results observed in a reference population. These intervals are typically defined as the mean ±2 times the standard deviation (SD) or as the 2.5th and 97.5th percentiles [2]. It is the responsibility of each laboratory to establish its own RIs. These must be subject to periodic review or revision in the event of a change to an analytical or pre-analytical procedure [3].

RIs can be adopted from the existing literature or *in vitro* diagnostic reagents’ manufactures. Furthermore, RIs can be calculated in a clinical laboratory using direct methods, which are based on studies performed on a representative sample of the healthy population, or through indirect methods [2], which are based on the analysis of a large volume of data from the results of analyses of patient samples [4].

In 2005, the *International Federation of Clinical Chemistry and Laboratory Medicine* (IFCC) established the Committee on Reference Intervals and Clinical Decision Values (C-RIDL) and in 2008 the *Clinical and Laboratory Standard Institute* (CLSI) published an official protocol for the preparation of RIs by direct methods [2], based on studies carried out in a representative cohort of the healthy population. A total of 120 healthy individuals are required per partition with data stratified according to age and sex, among other variables. For smaller sample sizes, robust method calculations are recommended, although the document acknowledges that these may yield wider confidence intervals. The statistical analysis of these data is relatively straightforward. Nevertheless, the direct methods to obtain RIs present significant limitations due to necessity of recruitment of a representative number of random individuals and the pre-analytical processing of samples obtained, which must accurately reflect the processing circuit of the routine laboratory. The definition of a healthy population is a complex process. Patient selection must be conducted during the medical consultation through anamnesis and a questionnaire on healthy habits and pathological history [5]. In some cases, obtaining samples is exceedingly challenging, particularly when the type of specimen or the patient population may pose a significant obstacle; examples thereof are cerebrospinal fluid samples or pediatric populations.

In contrast, indirect methods do not necessitate prior recruitment and are based on the analysis of a substantial volume of data obtained from the laboratory information system (LIS) database. These methods utilize data from primary care patients, employ algorithms to exclude potentially pathological data, and complex statistical studies that are performed to obtain a final sample for calculating RIs [6], [7], [8]. In 2019, the IFCC C-RIDL published recommendations that promoted an international effort to develop new RIs calculation protocols using indirect methods [9].

The C-RIDL recommends that each clinical laboratory should calculate its own RIs and perform a periodic review. One of the reasons for reviewing RIs is to accommodate modifications to an analytical or pre-analytical procedure. In April 2022, the Clinical Laboratories of the Vall d’Hebron University Hospital implemented a change in the analytical platform utilized in the automated biochemistry process from AU5800 (Beckman Coulter^®^) to Atellica (Siemens^®^). Initially, the RIs adopted for the new analytical platform were those recommended by the manufacturer. However, a higher-than-expected proportion of pathological results was subsequently observed for some biological analytes such as esterified bilirubin. Consequently, it became necessary to re-evaluate and calculate the own RIs, in this case, using an indirect approach from the reference population.

In light of these considerations, the objective was to establish a methodology based on preselection of patient samples for the calculation of RIs by indirect approach, with the aim of minimizing the proportion of pathological results and the necessary sample size.

## Materials and methods

This is a prospective study in which patients fulfilling inclusion criteria were recruited from the routine primary care to achieve a high proportion of healthy individuals.

### Sample management and patient selection

Preanalytical and analytical manipulation of serum samples was performed following our usual routine without extra management, as described in previous studies [10].

**Figure 1:**
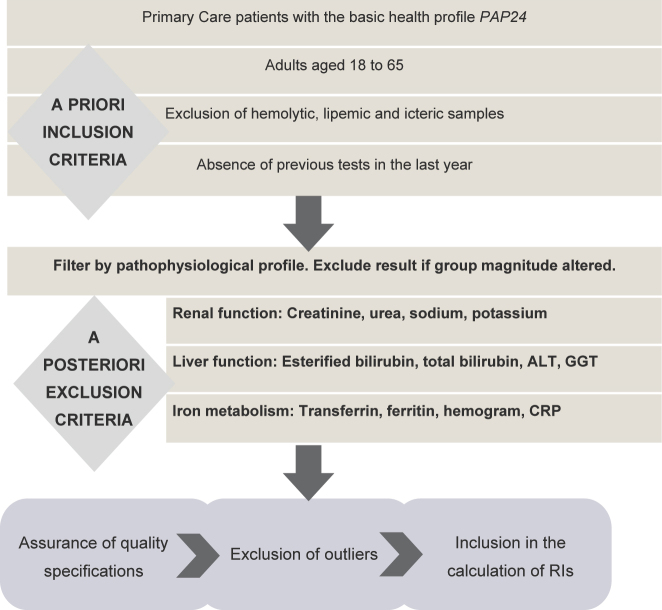
Workflow with the steps followed during the project.

To emulate a presumed healthy cohort, we applied *a priori* and *a posteriori* laboratory-based filters. Firstly, samples with any serum indices different from 0 were excluded (hemolysis >0.03 mmol/L hemoglobin, lipemia >0.45 mmol/L Intralipid^®^, icterus >23.94 μmol/L bilirubin). Secondly, requests had to be from patients aged 18–65 years with no prior laboratory results in the previous 12 months, for whom only the Basic Health Study profile (PAP24) was requested. Thirdly, to minimize latent pathology, we excluded records with associated-test results outside our laboratory reference intervals (Supplementary Table 1) for each pathophysiological profile (Figure 1).

The study included a total of 4,951 clinical requests, distributed across two recruitment periods: (1) April to June 2023 and (2) November 2023 to February 2024.

### Analytes studied

We targeted serum creatinine, esterified bilirubin, ferritin, and transferrin for RI estimation. For this purpose, each analyte was added to eligible routine requests as a non-reportable (“fictitious”) test, exclusively for study use and not included in clinical reports.

The protocol for the extension of requests, sample processing and data extraction was endorsed by the Vall d’Hebron Hospital Ethics Committee PR(AG)345/2022 (approved on October 18, 2022). This study was conducted in accordance with the principles of the Declaration of Helsinki and Spanish and European laws and regulations.

### Measurement procedures

The measurement procedures for the tests studied, as well as the units and traceable materials, are described in Supplementary Table 1.

### Quality assessment

To ensure the integrity and accuracy of the data, monthly results of external quality assurance programs, tailored to each analyte, were examined. The outcomes of the control materials obtained in the laboratory on a monthly basis were contrasted with the mean value calculated for each laboratory engaged in the program, employing the identical methodology and analytical system. If the result fell within the range ±2 times the SD of the participating laboratories, the data for the particular month and analytes were deemed acceptable. Else, the data from the specific analyte, month and instrument were excluded.

Conversely, the imprecision, bias and total error of all quantities were calculated using the Bio-Rad^®^ Unity Real Time program for inter- and intra-laboratory comparison of internal quality controls. The measurement of uncertainty and imprecision, bias and total error should not exceed the established specifications of the laboratory, which are based on biological variation [11].

To assess the analytical stability over time, the variability between different batches and seasonal changes, as well as daily moving averages, were calculated during the years 2023 and 2024 with the results of requests from Primary Care. Daily averages were calculated in series of 200 results and were visually compared with the average for the month and the average for the whole year.

### Calculation of reference values and statistical analysis

The statistical analysis for the calculation of RIs were carried out in different stages:

1. Study of data quality, ensuring compliance with clinical laboratory quality requirements and daily and monthly moving averages. Exclusion of results by month and magnitude when data quality was not ensured.
2. Exclusion due to associated test profile: renal profile, liver profile and iron metabolism profile described above.
3. Normality study using kurtosis and asymmetry tests.
4. Box-Cox transformation on the nonparametric distributions.
5. Study of partitions by sex using Mann-Whitney statistical test.
6. Detection of extreme values using Tukey’s statistical test.
7. Calculation of RIs using the 2.5 and 97.5 percentiles, as well as calculation of 95 % confidence intervals (CI). For one-tailed analytes, the same criteria were followed, ignoring the non-pathological limit.

### Sample size assessment

The minimum sample size (n) required for calculating RIs was analyzed using the method proposed by IFCC C-RIDL in 2010. The 95 % CIs of both the upper limit of normality (ULN) and the lower limit of normality (LLN) [12] were calculated with increasing n in chronological order of data collection, maintaining the overall age distribution. Sample sizes between 120 and 500 data were proposed. For each reference limit and magnitude, a factor was calculated from the equation:

$$Factor=\left(95\%\text{ CI of ULN or LLN}\right)/\left(2*{\text{SD}}_{\text{BI}}\right)$$

SD _BI_= Between individuals standard deviation.

### Evaluation of the results

To evaluate the statistical robustness of the RIs, we calculated both the total RI width (ULN – LLN) and the 90 % CI width for each limit. According to CLSI EP28-A3c recommendations, the statistical power of an RI is considered acceptable when the 90 % CI width of each limit is less than one-third of the total RI width [2].

Differences between the locally estimated RIs and those provided by the manufacturer were evaluated using the Bias Ratio (BR) method defined by Ozarda et al. [13].

This approach quantifies how far each limit (lower or upper) of the new RI deviates from the midpoint of the comparator interval, relative to the reference standard deviation (SD).

To evaluate potential differences, BR values were calculated for the lower limit (LL), midpoint (Me), and upper limit (UL) according to Eq. 1:

$$BR_{LL}=\frac{LL-LL_{0}}{SD_{RI}},BR_{Me}=\frac{Me-Me_{0}}{SD_{RI}},BR_{UL}=\frac{UL-UL_{0}}{SD_{RI}};\;SD_{RI}=\frac{UL_{0}-LL_{0}}{3.92}$$

which is the calculation of the bias ratio. LL; lower limit for the RIs to be evaluated, Me; central point of the reference interval, UL; upper limit. The designation LL _0_, Me _0_ and UL _0_ pertain to the RIs currently utilized in the laboratory settings. SD _RI_; standard deviation of the reference population.

As with the IFCC C-RIDL, the criterion of BR>|0.375| was adopted to indicate a significant difference between the intervals being compared [13]. RIs estimated from both recruitment periods were first compared with each other to verify internal consistency. Subsequently, the combined RIs obtained from full data set was compared against the manufacturer’s RIs.

### External validation of calculated reference values

The calculation of the pathological outcomes (flagging rates, FR) was performed by applying the calculated RIs to an independent dataset of primary care patients. The data collection periods for creatinine and esterified bilirubin were between January and March 2023 and between February and May 2024; while the collection period for transferrin and ferritin was between January and October 2023. Results are presented as the percentage of individuals whose values fall outside the reference intervals.

## Results

A total of 1,745 patients were included for creatinine, 1,362 for esterified bilirubin, 1,069 for ferritin and 1,131 for transferrin. The summary of included data distributed by age and sex for all the analytes is shown in Table 1.

**Table 1:** Number of participants who met the inclusion criteria distributed by age and sex.

Magnitude	Age, years	Total	Women	Men
Creatinine		1745	951	794
	18–35	30.3 % (n=528)	29.3 % (n=279)	30.1 % (n=249)
	36–45	26.1 % (n=455)	25.9 % (n=246)	25.2 % (n=209)
	46–55	25.0 % (n=437)	27.0 % (n=257)	21.7 % (n=180)
	56–65	13.5 % (n=325)	17.8 % (n=169)	18.8 % (n=156)
Esterified bilirubin		1,362	713	649
	18–35	26.7 % (n=364)	29.5 % (n=210)	23.7 % (n=154)
	36–45	26.4 % (n=359)	26.1 % (n=186)	26.7 % (n=173)
	46–55	24.5 % (n=334)	23.1 % (n=165)	26.0 % (n=169)
	56–65	22.4 % (n=305)	21.3 % (n=152)	23.6 % (n=153)
Ferritin		1,069	577	492
	18–35	25.1 % (n=268)	22.5 % (n=130)	28.0 % (n=138)
	36–45	29.6 % (n=316)	31.4 % (n=181)	27.4 % (n=135)
	46–55	24.2 % (n=259)	24.1 % (n=139)	24.4 % (n=120)
	56–65	21.1 % (n=226)	22.0 % (n=127)	20.1 % (n=99)
Transferrin		1,131	611	520
	18–35	25.1 % (n=284)	22.4 % (n=137)	28.3 % (n=147)
	36–45	29.5 % (n=334)	30.9 % (n=189)	27.9 % (n=145)
	46–55	24.0 % (n=271)	23.9 % (n=146)	24.0 % (n=125)
	56–65	21.4 % (n=242)	22.7 % (n=139)	19.8 % (n=103)

**Figure 2:**
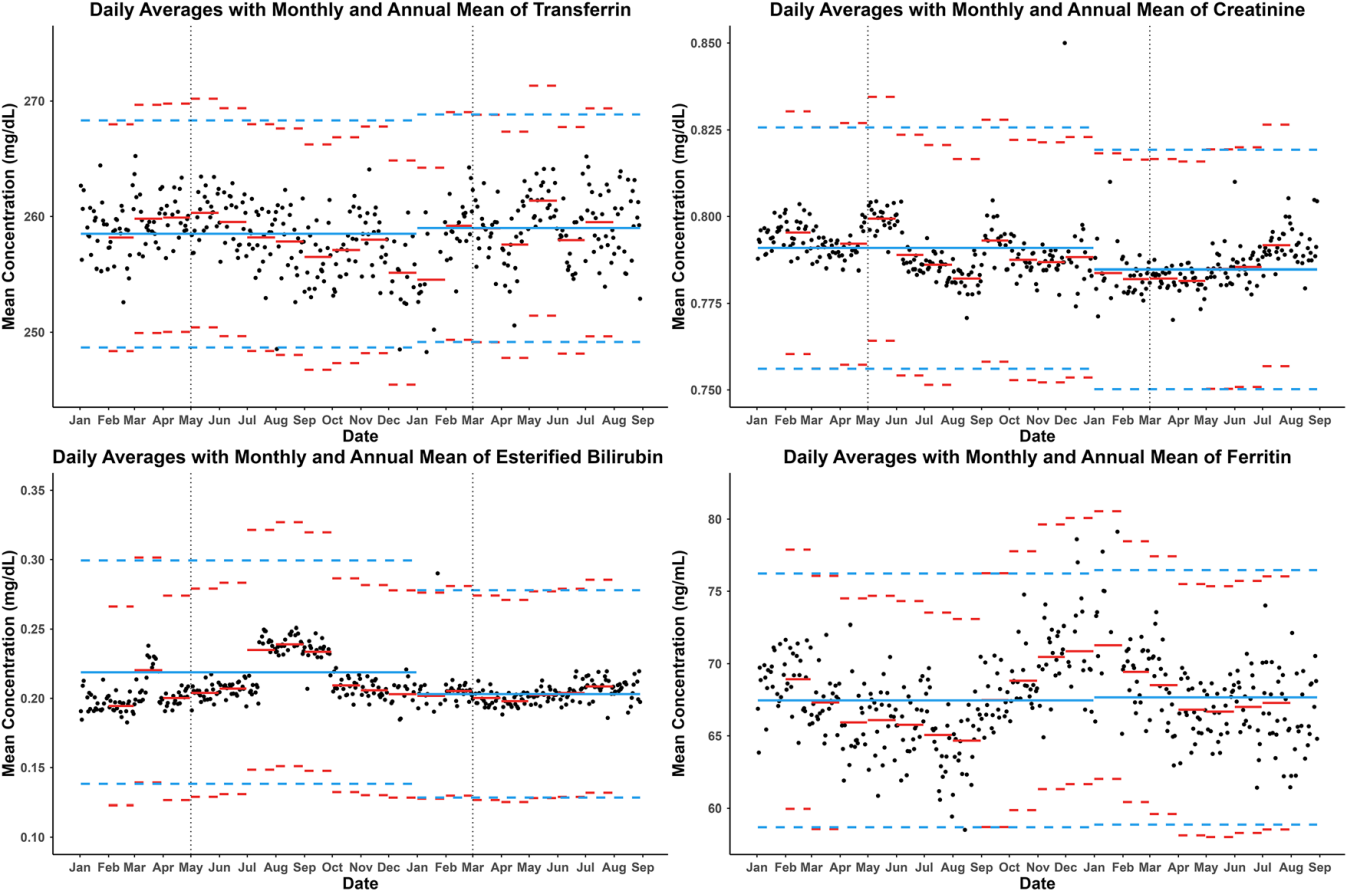
Daily averages of all primary care patients (black dot) for the different magnitudes studied. The monthly average (red) and the annual average (blue) are represented as lines and the biological variation of each magnitude as dotted lines.

All analytes met the quality criteria during the months of sample collection, and thus all data was included in the present study. The longitudinal variation observed in the graphs of daily, monthly and annual averages (Figure 2) was deemed acceptable.

The analytes exhibited a non-normal distribution, and RIs were calculated by sex based on results of Mann-Whitney test (p<0.05). The resulting RIs obtained are shown in Table 2.

**Table 2:** Reference values calculated with respect to the reagent manufacturer and BR results.

Biochemical parameter			First period	Second period	Global	Regent manufacturer	BR_LL_	BR_ME_	BR_UL_
Creatinine, mg/dL	F	RI	0.51–0.88	0.49–0.88	0.49–0.88	0.50–0.80 ​​[14]​	−0.01	0.07	0.15
		IC 95 % LLN	0.49–0.52	0.48–0.50	0.48–0.51				
		IC 95 % ULN	0.86–0.91	0.87–0.92	0.87–0.91				
	M	RI	0.68–1.15	0.67–1.13	0.67–1.14	0.60–1.10 ​​​​[14]​	0.07	0.06	0.05
		IC 95 % LLN	0.65–0.69	0.65–0.69	0.66–0.69				
		IC 95 % ULN	1.12–1.20	1.10–1.16	1.13–1.16				
Esterified bilirubin, mg/dL	F	RI	<0.32	<0.32	<0.32	<0.30 ​​​​[15]​	0.00	0.03	0.07
		IC 95 % LLN	NA	NA	NA				
		IC 95 % ULN	0.27–0.35	0.30–0.34	0.30–0.34				
	M	RI	<0.35	**<**0.37	<0.36		0.00	0.10	0.20
		IC 95 % LLN	NA	NA	NA				
		IC 95 % ULN	0.33–0.36	0.35–0.40	0.35–0.39				
Ferritin, ng/mL	F	RI	7–195			25–250 ​​​​[16]​	−0.07	−0.15	−0.22
		IC 95 % LLN	6–9						
		IC 95 % ULN	179–249						
	M	RI	27–428			25–400 ​​[16]​	0.00	0.04	0.07
		IC 95 % LLN	20–33						
		IC 95 % ULN	407–456						
Transferrin, mg/dL	F	RI	200–336			250–380 ​​[17]​	−0.16	−0.15	−0.14
		IC 95 % LLN	189–204						
		IC 95 % ULN	325–348						
	M	RI	192–311			215–365 ​​[17]	−0.07	−0.13	−0.19
		IC 95 % LLN	182–196						
		IC 95 % ULN	302–337						

F, female; M, male; RI, reference interval; LLN, lower limit of normality; ULN, upper limit of normality; BR_LL_, bias ratio for lower limit of the RIs; BR_ME_, bias ratio for central point of the RIs; BR_UL_, bias ratio for upper limit of the RIs.

Table 3 illustrates the factors obtained for each magnitude according to the sample size; CI widths decreased as n increased, with higher-skew analytes (creatinine, transferrin) requiring ≈500 cases to stabilize both limits, whereas near-Gaussian analytes (esterified bilirubin, ferritin) stabilized at ≈300–500, consistent with our factor analysis. An example for creatinine is represented in Supplementary Figure 1.

**Table 3:** Factor calculated for different sample sizes.

Biochemical parameter		n=120	n=200	n=300	n=400	n=500
Creatinine						
Female	LLN	0.46	0.36	0.22	0.24	0.21
	ULN	0.63	0.51	0.40	0.34	0.32
Male	LLN	0.49	0.29	0.19	0.14	0.10
	ULN	0.51	0.47	0.35	0.33	0.27
Esterified bilirubin						
Female	LLN	NA	NA	NA	NA	NA
	ULN	0.26	0.28	0.16	0.11	0.10
Male	LLN	NA	NA	NA	NA	NA
	ULN	0.36	0.15	0.08	0.19	0.16
Ferritin						
Female	LLN	0.31	0.29	0.28	0.22	0.22
	ULN	0.30	0.45	0.15	0.22	0.21
Male	LLN	0.21	0.30	0.32	0.25	0.23
	ULN	0.38	0.30	0.23	0.18	0.11
Transferrin						
Female	LLN	0.56	0.46	0.39	0.38	0.34
	ULN	0.45	0.42	0.33	0.27	0.27
Male	LLN	0.27	0.43	0.50	0.32	0.32
	ULN	0.35	0.32	0.44	0.46	0.41

LLN, lower limit of normality; ULN, upper limit of normality.

Regarding the evaluation of RIs obtained, the 90%CI width criterion was met for all analytes and partitions, confirming the adequacy of the sample size and the robustness of the estimated limits (Supplementary Table 2).

The reference intervals obtained at the two time points for creatinine and esterified bilirubin were compared using the BR calculation and no significant differences were found for BR_LL_ and BR_UL_ (BR<|0.375|). Additionally, the calculated RIs were compared with the previously utilized manufacturer-supplied values and no significant differences were found in any of the analytes or their partitions (Table 2).

**Figure 3:**
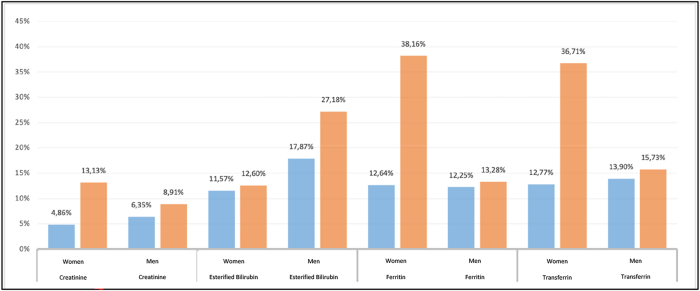
Flagging rates of the magnitudes studied. Blue bars represent the flagging rates based on the calculated reference intervals, while orange bars represent the flagging rates based on the manufacturer’s reference intervals.

The results of flagging rates obtained with the application of the RIs calculated in a population sample of 2024 are shown in Figure 3.

## Discussion

The utility of RIs provided by reagent manufacturers is often constrained by inherent limitations to the sample size used, the date of publication or the reference population selected. To solve the problem this study proposes an indirect approach for calculating reference intervals based on the use of routinely obtained laboratory samples, without prior recruitment of participants via inclusion and exclusion criteria, to obtain a reference sample from a population with a high proportion of healthy individuals [18], [19], [20], [21].

This design was conceived to achieve an adequate sample size maintaining a high proportion of non-pathological results for analytes that are infrequently requested in routine primary care panels. The indirect big-data methods – such as those implemented in the *refineR* algorithm [22] – are not optimal in this scenario, since these tests are typically ordered only in the presence of clinical suspicion. Consequently, despite the large volume of data stored in the LIS, the high prevalence of pathological results precludes their reliable use for indirect RI estimation. Therefore, our prospective recruitment is designed to ensure a biochemically healthy population, providing more robust and clinically meaningful RIs for these diagnostic-oriented analytes.

In addition, *a posteriori* exclusion criteria were used, predominantly based on the absence of pathological values in laboratory tests associated with the magnitude of interest [23]. The RIs obtained for esterified bilirubin, creatinine and ferritin in men were slightly higher than those currently in use, while in women the RIs for ferritin were slightly lower. These trends have been observed in recent articles carried out in populations like ours, in which indirect methods have also been used to calculate RIs [24], [25], [26]. Conversely, the reference interval has narrowed for the two transferrin partitions, mainly due to a decrease in the ULN. This change is compatible with the diagnosis of iron deficiency, where a pathological result for transferrin, higher than the ULN, would be expected, a fact that did not occur with the manufacturer’s RIs due to their higher ULN [27]. The lack of standardization of the measurement procedure could explain the differences observed in the RIs for ferritin and transferrin from different studies [26], 28], 29]. Standardization of the measurement procedures allows the calculation of RIs in multicenter studies, which could be a significant advantage in terms of optimizing resources and increasing sample size [4].

Regarding the stratification performed in this study, all the analytes exhibited notable disparities by sex, in accordance with previously published findings [28], [29], [30], [31].

Regarding the periodic review of RIs, no differences were observed between the two study periods. To date, there is no agreement on the necessity of periodic review and if so, on the appropriate frequency for recalculation. The IFCC does not prioritize this in any of the recommended direct and indirect methodologies, beyond recommending the collection of data over a period of at least 1 year to reveal circannual variations [9].

In accordance with CLSI EP28-A3c, we verified the statistical reliability of the reference limits by ensuring that the 90 % CI width for each limit remained below one-third of the overall RI width. This condition was satisfied in all analytes and partitions, supporting that the derived RIs were based on a sufficiently powered dataset. The fulfillment of this criterion indicates that sampling variability had a minimal influence on the final limits, reinforcing the stability and robustness of the intervals.

Although the BR metric did not indicate statistically significant changes vs. manufacturer RIs, FR decreased markedly when applying our locally derived intervals (e.g., transferrin and ferritin in women; esterified bilirubin in men), aligning FRs closer to the expected 5 % in a healthy reference population (Figure 3). This apparent discrepancy arises because BR primarily captures relative shifts in limits against the comparator midpoint, while FR reflects the interaction between those limits and the real-world distribution of test results in our catchment. Thus, even modest limit adjustments – below the BR threshold – can substantially reduce false-positive calls in a population whose distribution differs from that assumed by the manufacturer.

The percentage of pathological results or flagging rates calculated from the new RIs were found to be lower than those provided by the manufacturer for all the analytes and study partitions. According to the statistical model of RIs in which 95 % of the healthy population is within the intervals [4], 32], the percentage of FR should be adjusted to 5 %. In this sense, only the FR for creatinine have been adjusted to 5 %, given that magnitude is requested as a default in the basic health profile of primary care and the proportion of healthy individuals in the database is relatively high. The remaining analytes exhibited higher FRs, which is to be expected given that none of those are usually requested from primary care without associated symptoms. Consequently, the proportion of healthy individuals in this cohort is lower. However, for the three analytes and their respective partitions by sex, the percentage of false positives decreased. In women, this change was notable, with a decrease from 36.71 to 12.77 % in transferrin and from 38.16 to 12.64 % in ferritin. In men, the change was also significant, with a decrease from 27.18 to 17.87 % in esterified bilirubin. In conclusion, the adjustment of the RIs to the reference population has a considerable impact on healthcare centers, from the primary care physician to the specialist physician who requests the analysis, reviews the results, visits the patient, and prescribes a treatment or follows up in the event of a pathological result in the analysis. Additionally, it influences on the laboratory workload.

Currently, there is no indication as to the minimum sample size required for an RI calculation. However, to obtain robust results, it is recommended that a larger sample size be used [9]. The results demonstrated that a minimum sample size exceeding 120 samples is necessary, as recommended for direct methods [33].

While IFCC C-RIDL endorses ≥400 individuals per partition for robust indirect RIs [31], our empirical analysis showed that the minimum n depends on distributional asymmetry: analytes with higher skew (e.g., creatinine, transferrin) required n≥500 to stabilize the CI width at both limits, whereas near-Gaussian analytes (esterified bilirubin, ferritin) stabilized with n≈300–500 (Table 3). Thus, while our prospective approach deliberately limited n compared with retrospective big-data methodologies, it enhanced internal validity through controlled preanalytical conditions and continuous analytical QC, and still achieved precise limits for analytes with less skew. Despite a moderate sample size, our cohort represents a biochemically healthier population compared with large big-data studies. Indirect RIs studies based on massive datasets typically achieve higher n at the expense of including a substantial proportion of unrecognized pathological results. This is particularly relevant for analytes that are requested predominantly in a diagnostic (non-screening) context, such as ferritin, transferrin, or esterified bilirubin, where big-data approaches would be expected to include a higher fraction of pathological values and therefore yield biased interval limits.

This approach was proposed to obtain RIs specific to each participating country in a more reproducible manner [34]. These recommendations are only partially aligned with the findings, as it has been demonstrated that for analytes with greater asymmetry, an even larger sample size may be necessary. Given the lack of consensus on the requisite sample size for calculating RIs by indirect methods, it would be prudent to establish this parameter on a methodology-specific basis, with the aim of ensuring the most robust statistical approach. In general, indirect methods for calculating RIs are suitable when a large amount of data is available from a population with a high proportion of healthy individuals [35].

The RIs obtained in this study are intended for application in healthcare practice, aiming to support more accurate clinical decision-making, enhance patient outcomes and contribute to the optimization of healthcare system resources. Furthermore, the RIs estimated can be transferred/adapted to other laboratories whose reference population has similar demographics, provided that a verification process is performed.

This study has some weaknesses. Firstly, a limitation intrinsic to the methodology employed is the inability to review the medical records of all the patients included in the study or to administer a health and habits questionnaire, which may introduce a bias in the calculation of the RI if the data are not adequately filtered. Secondly, the sample size obtained for the study was deliberately limited to create a more cost-effective and sustainable method.

## Conclusions

The findings support the assertion that improved methodologies for determining RIs represent an effective and feasible alternative to conventional approaches. The mixed methodology we propose combines the advantages of the direct method, as the ability to obtain robust statistical results without requiring large datasets, with the strengths of indirect approaches. This allows sample handling under conditions similar to routine clinical workflows and enables the exclusion of certain participants based on analytical results. Furthermore, by obviating the need to recruit healthy subjects, this approach substantially reduces operational costs and enhances efficiency.

The adaptation of the RI to the demographic reference population has the effect of reducing the percentage of incorrect pathological results, which in turn implies a lower number of false positives. This results in a notable optimization of the workload in clinical laboratories and a more efficient utilization of health system resources, which positively impacts the comprehensive management of patients.

## Supplementary Material

Supplementary Material
