# Supplementary material for: Calculation of reference intervals using an indirect approach from laboratory database
Source: Adv Lab Med. 2026 Feb 9;7(1):34–42. doi: 10.1515/almed-2025-0088 (PMC12994705; doi:10.1515/almed-2025-0088)
Supplement: Supplementary file 1 — Supplementary Material [file j_almed-2025-0088_suppl_001.docx]

Supplemental Table 1. Laboratory magnitudes and parameters included in the study and measurement characteristics.

| Analyte | Units | Reference Intervals | Measurement procedure | Traceable material |
| --- | --- | --- | --- | --- |
| Alanine aminotransferase | UI/L | 10-49 | Photometry - NADH, by α -ketoglutarate addition | IRMM/IFCC-454 |
| Esterified bilirubin | mg/dL | <0.3 | Photometry - Oxidation with vanadate, pH 3 | SRM 916 (NIST) |
| Total bilirubin | mg/dL | 18-60y: 0.3-1.2  >60y: 0.2-1.1 | Photometry - Oxidation with vanadate, pH 10 | SRM 916 (NIST) |
| Creatinine | mg/dL | M: 0.6-1.1  F: 0.5-0.8 | Photometry - Enzymatic method (creatininase, sarcosine oxidase, peroxidase) | SRM 967 (NIST) |
| Ferritin | ng/mL | M: 25-400  F: 25-250 | Immunoturbidimetry - Latex microparticles coated with anti-ferritin antibody | WHO 3rd International Standard/IBSC 94/572 |
| Gamma-glutamyl transferase | UI/L | M: 7-73  F: 7-28 | Photometry - 5-amino-2-nitrobenzoate, by addition of L-γ-Glutamyl-3-Carboxy-4-Nitroanilide + glycylglycine | IRMM/IFCC-452 |
| Glucose | mg/dL | 74-106 | Photometry - Enzymatic method (hexokinase, G6PDH) | SRM 965a (NIST) |
| Potassium | mmol/L | 3.5-5.1 | Indirect Potentiometry - ALYTE Integrated Multisensor (IMT Na K Cl) | F-AES ^Δ^, which uses reference materials from NIST |
| C-reactive protein | mg/dL | <0.33 | Immunoturbidimetry - Latex microparticles coated with anti-PCR antibodies | ERMDA-470 (IRMM) |
| Sodium | mmol/L | 136-146 | Indirect Potentiometry - ALYTE Integrated Multisensor (IMT Na K Cl) | F-AES ^Δ^, which uses reference materials from NIST |
| Transferrin | mg/dL | M: 215-365  F: 250-380 | Immunoturbidimetry - PEG-enhanced human anti-transferrin antibody | CRM 470 (IFCC) |
| Urea | mg/dL | 19-49 | Photometry - Enzymatic method (urease, glutamate dehydrogenase) | SRM 912 and 909 (NIST) |

M, male; F, female; ^Δ^ Flame Atomic Emission Spectrophotometry (F-AES).

Supplemental Table 2. Relation of the 1/3 RI width and 90%CI width of the lower and upper limit of normality.

| **Biochemical Parameter** |  |  |  |  | **Width 1/3 RI** | **Width IC90%** |
| --- | --- | --- | --- | --- | --- | --- |
| **Creatinine** | **F** | **RI** | **0.49-0.88** |  | **0.13** |  |
|  |  | IC90% LLN | 0.48-0.50 |  |  | 0.02 |
|  |  | IC90% ULN | 0.87-0.90 |  |  | 0.03 |
|  | **M** | **RI** | **0.67-1.14** |  | **0.16** |  |
|  |  | IC90% LLN | 0.66-0.69 |  |  | 0.03 |
|  |  | IC90% ULN | 1.13-1.16 |  |  | 0.03 |
| **Esterified bilirubin** | **F** | **RI** | **<0.32** |  | **0.11** |  |
|  |  | IC90% LLN | NA |  |  |  |
|  |  | IC90% ULN | 0.30-0.33 |  |  | 0.03 |
|  | **M** | **RI** | **<0.36** |  | **0.12** |  |
|  |  | IC90% LLN | NA |  |  |  |
|  |  | IC90% ULN | 0.35-0.38 |  |  | 0.03 |
| **Ferritin** | **F** | **RI** | **7-195** |  | **62.67** |  |
|  |  | IC90% LLN | 6-9 |  |  | 3 |
|  |  | IC90% ULN | 181-236 |  |  | 55 |
|  | **M** | **RI** | **27-428** |  | **133.67** |  |
|  |  | IC90% LLN | 20-32 |  |  | 12 |
|  |  | IC90% ULN | 402-440 |  |  | 38 |
| **Transferrin** | **F** | **RI** | **200-336** |  | **45.33** |  |
|  |  | IC90% LLN | 190-204 |  |  | 14 |
|  |  | IC90% ULN | 324-343 |  |  | 19 |
|  | **M** | **RI** | **192-311** |  | **39.67** |  |
|  |  | IC90% LLN | 184-196 |  |  | 12 |
|  |  | IC90% ULN | 302-322 |  |  | 20 |

F, female; M, male; RI, reference interval; LLN, lower limit of normality; ULN, upper limit of normality

Supplemental Figure 1. Representation of the variation of the factor with increasing N for creatinine. The arrow indicates the minimum sample size required for each subgroup.


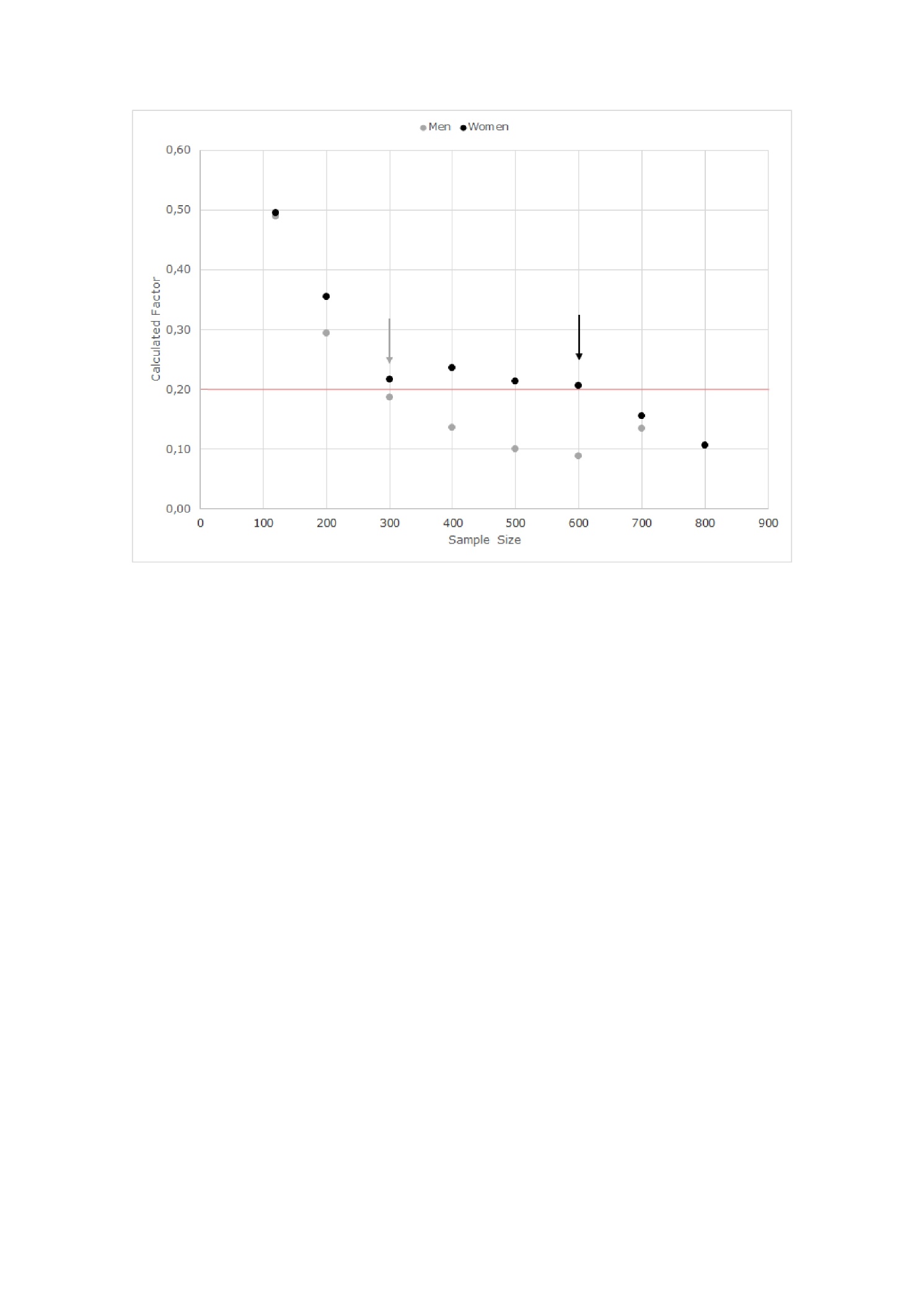


Men Women

Calculated Factor

Sample Size
